# Guillain-Barre syndrome in patients receiving chimeric antigen receptor T-cell therapy: an individual participant data meta-analysis

**DOI:** 10.3389/fneur.2025.1704826

**Published:** 2025-11-13

**Authors:** Kathleen M. Kilroe, Jamie E. Clarke, Phoebe Ann, Noriko Salamon, Jay Acharya

**Affiliations:** 1 David Geffen School of Medicine, University of California Los Angeles (UCLA), Los Angeles, CA, United States; 2 Department of Diagnostic Radiology, University of California Los Angeles (UCLA), Los Angeles, CA, United States

**Keywords:** Guillain-Barré syndrome, CAR T-cell therapy, neurotoxicity, cytokine release syndrome, immune effector cell-associated neurotoxicity syndrome, cranial nerve palsy

## Abstract

Chimeric antigen receptor (CAR) T-cell therapy has revolutionized the treatment of hematologic malignancies but is increasingly associated with unique neurotoxic complications. While cytokine release syndrome (CRS) and immune effector cell-associated neurotoxicity syndrome (ICANS) are well-characterized, Guillain-Barré syndrome (GBS) remains a rare and underrecognized adverse event. This PRISMA-guided systematic review, supplemented by a novel case report, outlines the clinical, radiographic, and diagnostic characteristics of GBS following CAR T-cell therapy. A total of 10 cases were evaluated, including a case from our institution involving a 30-year-old male with high-grade B-cell lymphoma who developed GBS with lasting neurological deficits despite treatment. Across reported cases, the onset of GBS ranged from 5 to 78 days following CAR T-cell infusion and was frequently preceded by CRS. Notably, 60% of patients exhibited facial nerve involvement, with cranial neuropathies often preceding peripheral symptoms, an atypical presentation that differs from classic GBS. Radiographic imaging often demonstrated facial nerve enhancement, while cerebrospinal fluid analysis revealed albuminocytologic dissociation with mild pleocytosis. Although intravenous immunoglobulin (IVIG) was the mainstay treatment, clinical responses were limited, raising questions about pathophysiology. Unlike classic GBS, which is typically antibody-mediated, CAR T-cell–associated GBS may stem from non-specific immune activation and cytokine-driven bystander injury. This review suggests CAR T-cell–related GBS may represent a distinct clinical entity with unique radiologic findings. Early recognition and further mechanistic investigation are essential to guide effective management.

## Introduction

Chimeric antigen receptor (CAR) T-cell therapy represents an innovative form of cell-based immunotherapy, offering significant therapeutic potential for hematologic malignancies, particularly among patients with relapsed or refractory disease. This modality involves the ex vivo genetic modification of autologous T cells to express synthetic receptors targeting tumor associated antigens, followed by reinfusion into the patient (1). In cases of refractory diffuse large B-cell lymphoma, CAR T-cell therapy has been associated with complete remission rates of approximately 47–51%, compared to just 7–26% with conventional salvage chemotherapy (2–4). Currently, six CAR T-cell therapies are approved by the United States Food and Drug Administration (FDA) for various hematologic malignancies, including tisagenlecleucel, axicabtagene ciloleucel, lisocabtagene maraleucel, brexucabtagene autoleucel, idecabtagene vicleucel, and ciltacabtagene autoleucel (5–10). While CAR T-cell therapy offers a potentially curative option for otherwise treatment-resistant cancers, it is also associated with more frequent and severe neurotoxic effects compared to standard chemotherapy (11, 12).

The two most commonly reported adverse events following CAR T-cell therapy are cytokine release syndrome (CRS) and immune effector cell-associated neurotoxicity syndrome (ICANS) (1). CRS results from the robust activation of CAR T cells upon encounter with tumor cells, leading to a surge in pro-inflammatory cytokines, including interleukin 6 (IL-6), interferon gamma (IFNγ), interleukin 1 (IL-1), and tumor necrosis factor alpha (TNF-α), that drive systemic inflammation. This excessive immune response results in clinical manifestations such as fever, chills, nausea, and capillary leak that can lead to vasodilatory shock and end-organ dysfunction (13, 14). The incidence of CRS following CAR T-cell therapy has been reported in up to 77–93% of patients, although this varies depending on patient characteristics, disease type, and the specific CAR T-cell product used (7, 15, 16).

ICANS, whose exact pathophysiology remains unclear, is thought to result from endothelial activation in CRS, causing disruption of the blood–brain barrier and off-target immune effects resulting in neuronal injury. Permeability of the neurovascular unit leads to cytokine infiltration into the central nervous system (CNS), resulting in astrocyte injury, increased cerebrospinal fluid protein, and penetration of T cells into the CNS (17–20). Clinically, ICANS may present with confusion, headaches, and aphasia (21). Another potential manifestation of ICANS is dysgraphia, or the impaired ability to produce legible handwriting (22, 23). In severe cases, patients may even experience seizures or cerebral edema (21). Brain magnetic resonance imaging (MRI) findings are frequently normal; however, abnormalities such as T2/FLAIR hyperintensities in the bilateral thalami, external capsules and the dorsal brainstem, as well as leptomeningeal enhancement, are occasionally observed (24, 25). The incidence of ICANS has been reported in up to 27% of patients, with variability based on numerous clinical and therapeutic factors (26). While cytokine release syndrome and immune effector cell-associated neurotoxicity syndrome are the most common and well-characterized neurologic complications of CAR T-cell therapy, a broader spectrum, including parkinsonism, acute quadriparesis, and Guillain-Barré syndrome (GBS), has also been reported. GBS, a rare but potentially life-threatening acute inflammatory demyelinating polyneuropathy, poses unique diagnostic and therapeutic challenges in the setting of CAR T therapy. Though typically triggered by infections such as *Campylobacter jejuni*, or less commonly by vaccinations or surgery, GBS following CAR T-cell therapy has been described in isolated case reports and small series. Proposed mechanisms of GBS following CAR T-cell therapy include cytokine-driven inflammation, autoreactive T-cell–mediated peripheral nerve injury, and loss of immune tolerance. CAR T-cell therapy leads to a surge of inflammatory cytokines which can drive the expansion or activation of autoreactive CD4+ and CD8+ T cells that target myelin antigens in peripheral nerves. Similarly, this cytokine storm can disrupt immune homeostasis and cause bystander damage to peripheral nerves. CAR T-cell therapy can also lead to B-cell depletion followed by immune reconstitution, with unmasking or triggering the production of autoantibodies directed against components of peripheral nerves (27, 28). While biologically plausible, the evidence is limited to case reports and lacks definitive mechanistic studies, such as immune profiling, autoantibody detection, or nerve histopathology, making causality difficult to establish. Despite these limitations, the known immune-modulating effects of CAR T therapy and temporal associations in reported cases support its consideration as a plausible and clinically relevant trigger for GBS.

Clinically, GBS typically presents as progressive, symmetric, ascending muscle weakness, often accompanied by sensory disturbances, areflexia, dysautonomia, and, in severe cases, respiratory compromise (28, 29). Subtypes include acute inflammatory demyelinating polyradiculoneuropathy (AIDP), acute motor axonal neuropathy (AMAN), acute motor and sensory axonal neuropathy (AMSAN), Miller Fisher syndrome (MFS), and the pharyngeal–cervical–brachial variant. The diagnosis is primarily clinical but may be supported by cerebrospinal fluid analysis demonstrating albuminocytologic dissociation and by electrodiagnostic studies consistent with axonal damage (28, 30). Despite well-established diagnostic and treatment protocols for classic GBS, CAR T–associated GBS remains poorly characterized due to its rarity. Here, we present a representative case from our institution and review the clinical and radiographic findings reported in the literature.

## Methods

This review conforms to the “Preferred Reporting Items for Systematic Reviews and Meta-Analyses” (PRISMA) statement.

### Search strategy and selection criteria

#### Identification

**Figure 1:**
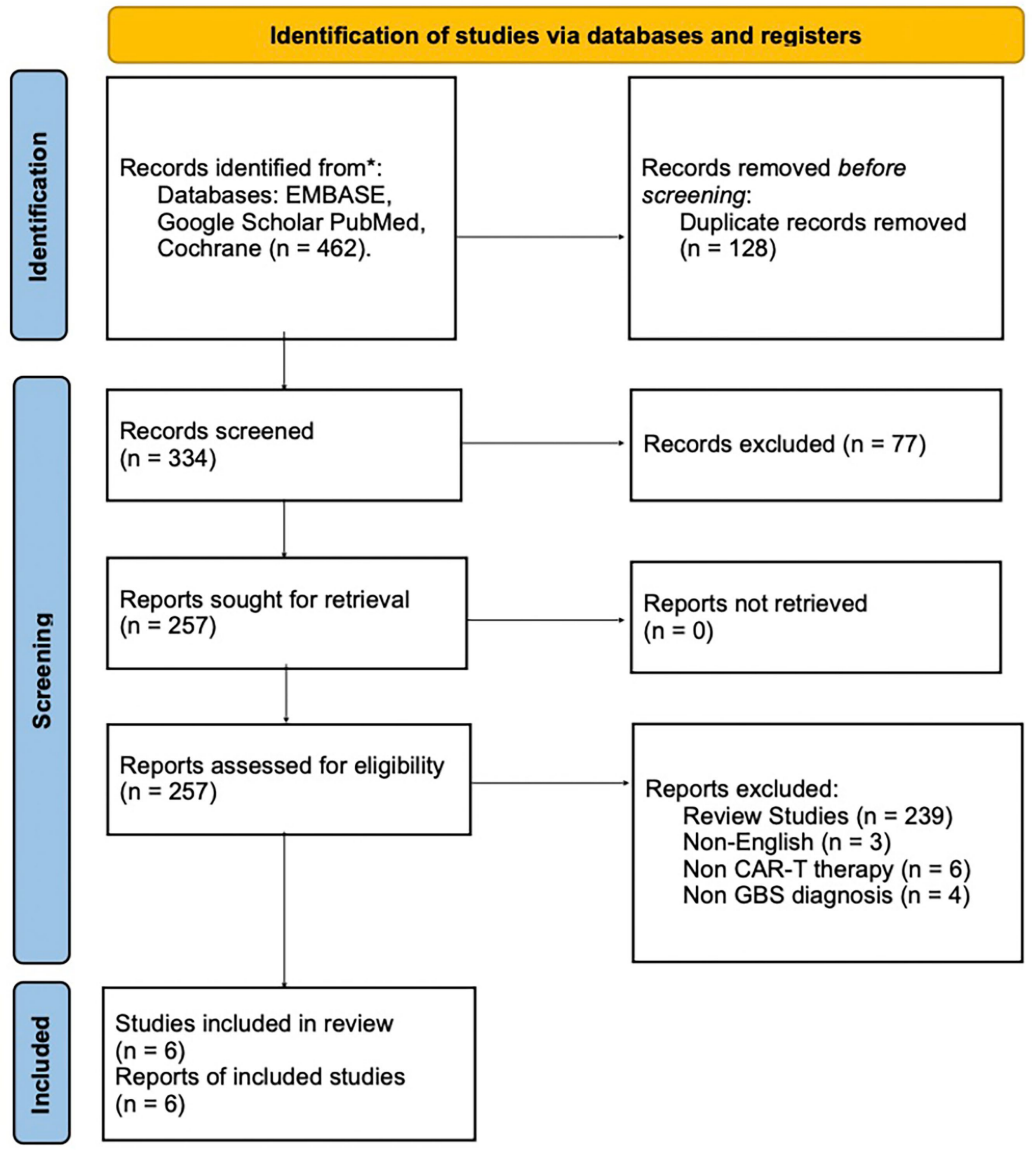
PRISMA 2020 flow diagram summarizing the literature search and selection process for GBS cases following CAR T-cell therapy. Flowchart illustrating the study selection process in accordance with PRISMA 2020 guidelines. It details the number of records identified through electronic database searches, duplicates removed, and the screening steps conducted prior to full-text eligibility assessment.

A systematic search of PubMed, Google Scholar, EMBASE, and Cochrane reviews was conducted using relevant keywords and phrases. The search covered studies from inception to May 1, 2025. The following keywords were used: “CAR T-cell AND GBS” OR “CAR T-cell therapy Guillain Barre Syndrome Case Report” OR “Guillain Barre Syndrome CAR T-cell therapy” OR “Kymriah tisagenlecleucel CAR T-cell therapy Guillain Barre Syndrome Case Report” OR “Yescarta axicabtagene ciloleucel CAR T-cell therapy Guillain Barre Syndrome Case Report” OR “Breyanzi lisocabtagene maraleucel therapy Guillain Barre Syndrome Case Report” OR “Tecartus brexucabtagene autoleucel therapy Guillain Barre Syndrome Case Report” OR “Abecma idecabtagene vicleucel therapy Guillain Barre Syndrome Case Report” OR “Carvykti ciltacabtagene autoleucel therapy Guillain Barre Syndrome Case Report.” The search timeframe was limited to publications from 2017 to the present, as CAR T-cell therapy first received FDA approval in 2017. The search yielded 445 abstracts from Google Scholar, 12 from PubMed, 5 from Embase, and 0 from Cochrane reviews (Figure 1).

#### Screening and eligibility

The articles and scholarly papers were reviewed by author KK. Any uncertainties were discussed and resolved in consultation with the second author (JC). To maintain data integrity, all findings were recorded in Microsoft Excel, and duplicates were eliminated through list comparisons. Studies were included if they were case reports or case series linking clinically significant Guillain-Barré syndrome (GBS) to CAR T-cell therapy, and if full-text articles were available. Exclusion criteria included review articles, clinical trials, translational research, letters to the editor, non-English articles, and studies lacking detailed case report information.

A comprehensive database search initially identified 462 abstracts. Of these, 128 were duplicates already encountered in the literature. An additional 77 abstracts were excluded based on irrelevant titles or content, such as studies discussing GBS in the context of COVID-19, neuropathies associated with vincristine use, and other unrelated topics. Following the established screening and eligibility criteria, 239 scholarly papers were removed for being reviews, clinical trials, or translational research. Thirteen more articles were excluded because they were either not in English or were case reports that did not meet the inclusion criteria. Ultimately, five case reports and one case series were identified, in which four of the patients met the inclusion criteria. The screening process is depicted in Figure 1, using the PRISMA flow diagram.

#### Data extraction

The following data points were collected from the included cases and recorded in a data extraction report: age, sex, hematologic malignancy and stage, prior chemotherapy history, type of CAR T-cell therapy used, time to onset of symptoms following CAR T-cell therapy, peripheral nerve involvement, cranial nerve involvement, type of imaging performed, radiographic imaging findings, cerebrospinal fluid analysis, nerve conduction study report, CRS diagnosis and timeline, ICANS diagnosis and timeline, intubation status, and use of intravenous immunoglobulin (IVIG) in treatment and dosing.

#### Case presentation

*Case 1:* The patient is a 30-year-old male diagnosed with high-grade large B-cell non-Hodgkin lymphoma, for which he received Yescarta CAR T-cell therapy for refractory disease. Initially, he presented to an outside hospital with persistent abdominal pain, and a lymph node biopsy confirmed the diagnosis of high-grade B-cell lymphoma, which had transformed from lymphoplasmacytic lymphoma (MYD88- and CXCR4-negative). The patient initially underwent two cycles of the DA-EPOCH-R regimen with intrathecal methotrexate; however, follow-up PET-CT scans revealed persistent disease (Lugano classification stage 5). Additionally, he developed fingertip paresthesia during chemotherapy, which was attributed to vincristine-induced neuropathy. This moderate neuropathy resolved within 2 months, prior to the initiation of CAR T-cell therapy. Given the radiologic findings, the patient underwent a bridging regimen of DA-EPOCH-R, omitting vincristine due to the prior neuropathy, followed by CAR T-cell therapy. The CAR T-cell therapy was administered after lymphodepletion with fludarabine and cyclophosphamide. The patient developed neutropenia following lymphodepletion, which persisted for 4 months before resolving.

**Figure 2:**
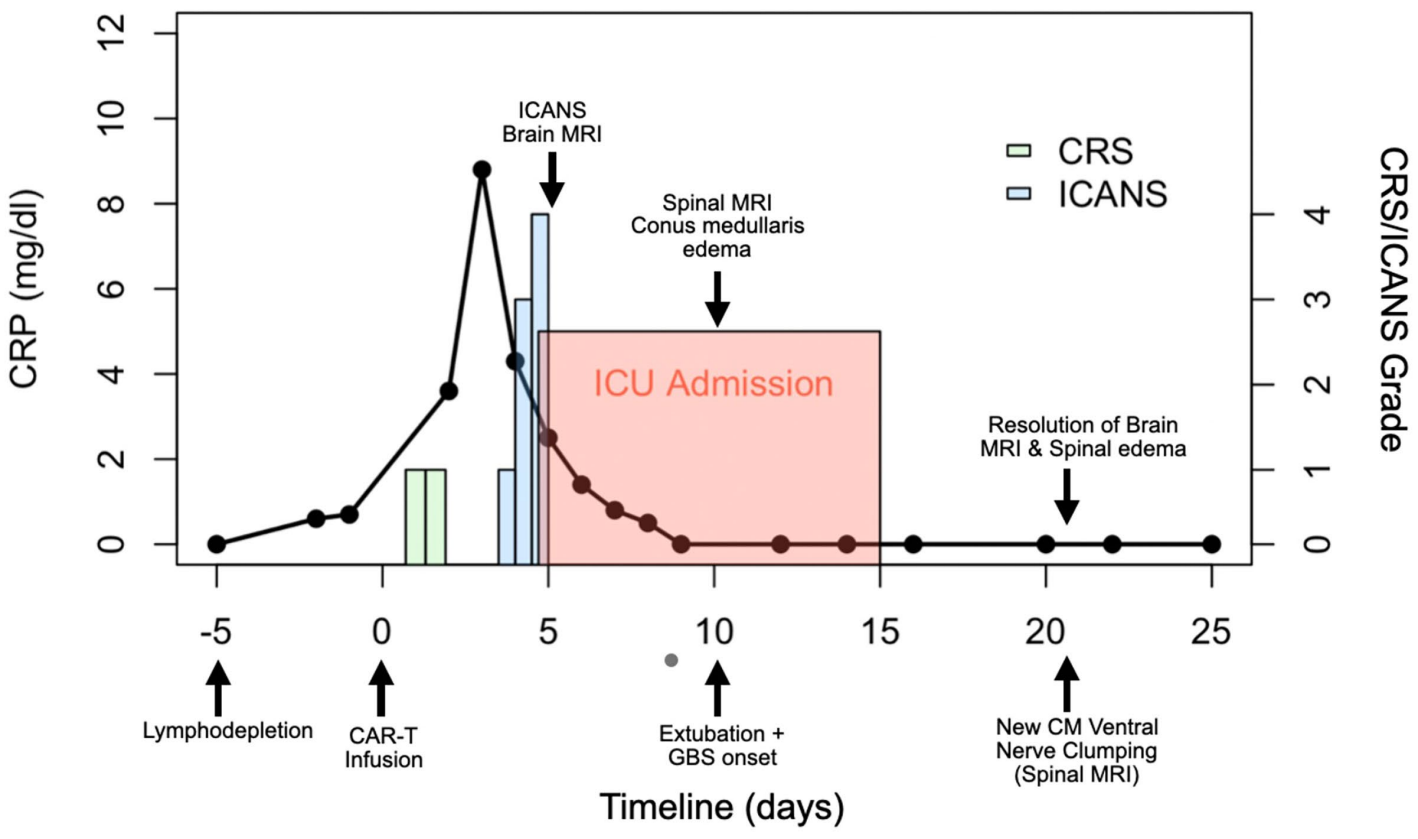
Clinical course timeline of symptoms and key findings in the reported case. Day 0 marks the infusion of CD19-targeted CAR T-cell therapy. Neurological progression and corresponding imaging findings are annotated at their respective time points. The left panel shows the patient’s C-reactive protein (CRP) levels over time, while the right panel displays the onset and severity of cytokine release syndrome (CRS) and immune effector cell-associated neurotoxicity syndrome (ICANS), categorized by grade. CM = conus medullaris.

**Figure 3:**
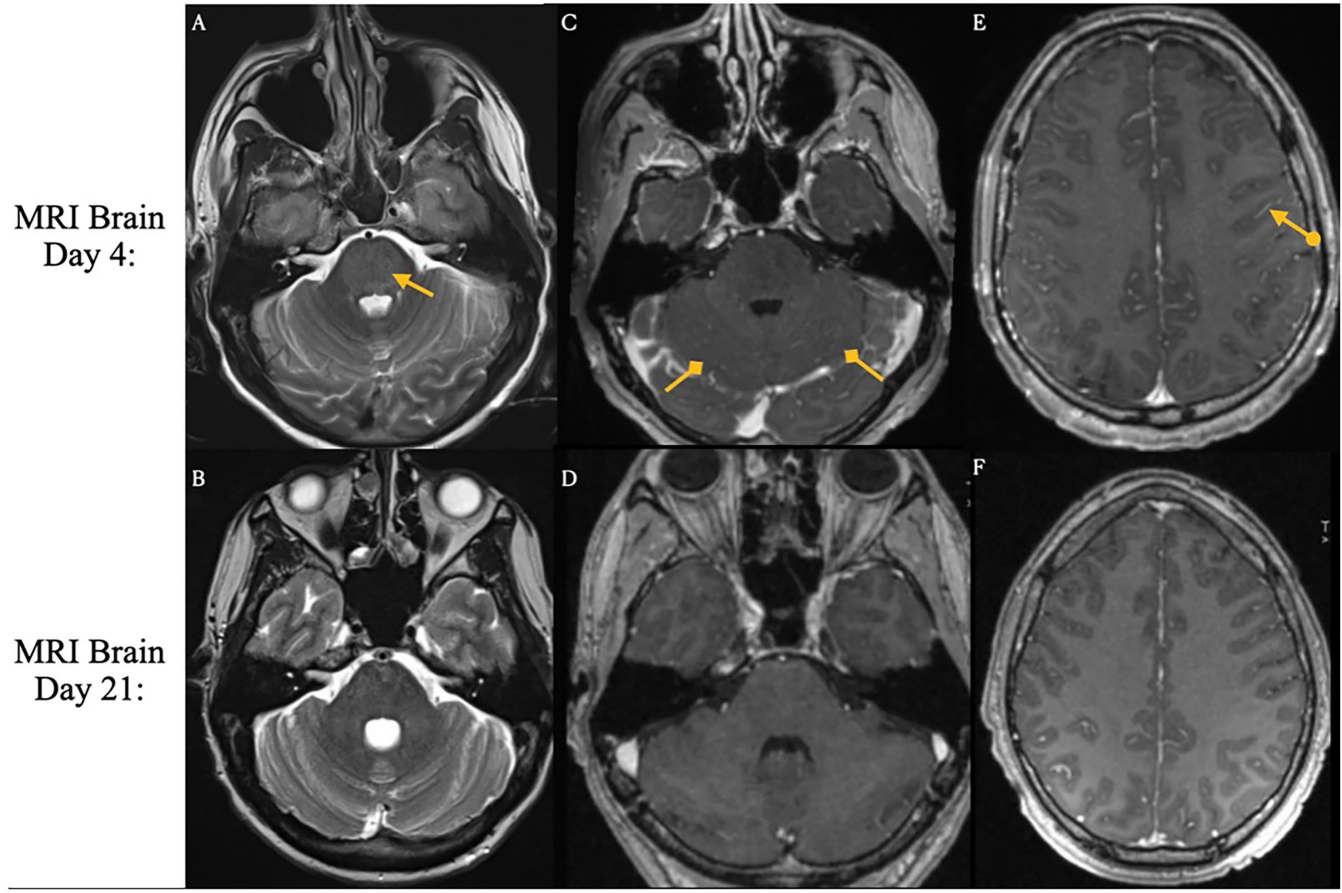
Brain magnetic resonance imaging on days 4 and 21 following CAR T-cell infusion. ICANS-associated brain MRI findings. T2 and FLAIR sequences reveal hyperintensity throughout the cortex and pons, accompanied by diffuse leptomeningeal enhancement. Images were acquired pre-treatment (Day 4 Panels **A,C,E**) and post-treatment (Day 21. Panels **B,D,F**) following steroid and Anakinra therapy.

**Figure 4:**
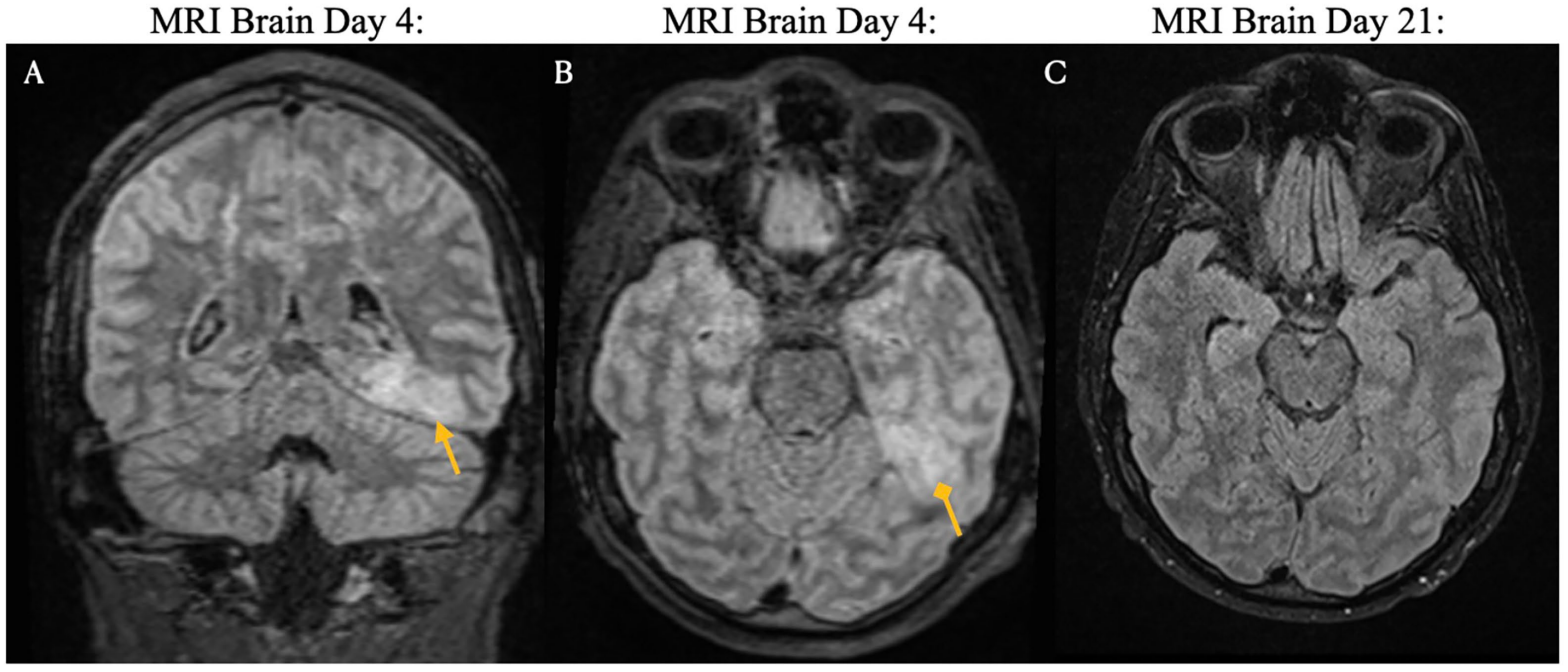
Brain magnetic resonance imaging on days four and 21 post-CAR T-cell infusion. ICANS-associated brain MRI findings showing focal FLAIR hyperintensity in the left mesial temporal lobe (Panels **A,B**), which may reflect recent seizure activity. Images from pre-treatment (Day 4: Panels **A,B**) and post-treatment (Day 21: Panel **C**) with steroids and Anakinra demonstrate resolution of the lesion.

The clinical timeline for this patient is illustrated in Figure 2. One day after CAR T-cell therapy, the patient developed persistent fevers consistent with grade 1 CRS, and was treated with tocilizumab, resulting in resolution of the fever. However, 3 days later, he experienced recurrent grade 1 CRS with fever and grade 1 immune effector cell-associated neurotoxicity syndrome. The patient’s condition rapidly deteriorated, progressing to grade 3 ICANS (aphasia) and subsequently to grade 4 ICANS, characterized by seizure activity. On day five, he was intubated for airway protection and transferred to the intensive care unit (ICU). MRI of the brain revealed diffuse leptomeningeal enhancement, widespread T2 and FLAIR hyperintensities throughout the cortex and pons (Figure 3), and focal FLAIR hyperintensity in the left mesial temporal lobe (Figure 4), potentially related to recent seizure activity. These neuroimaging findings were consistent with ICANS-associated changes. The patient was started on dexamethasone and anakinra for the management of ICANS-related neurotoxicity, and by the following day, there was a marked reduction in leptomeningeal enhancement.

**Figure 5:**
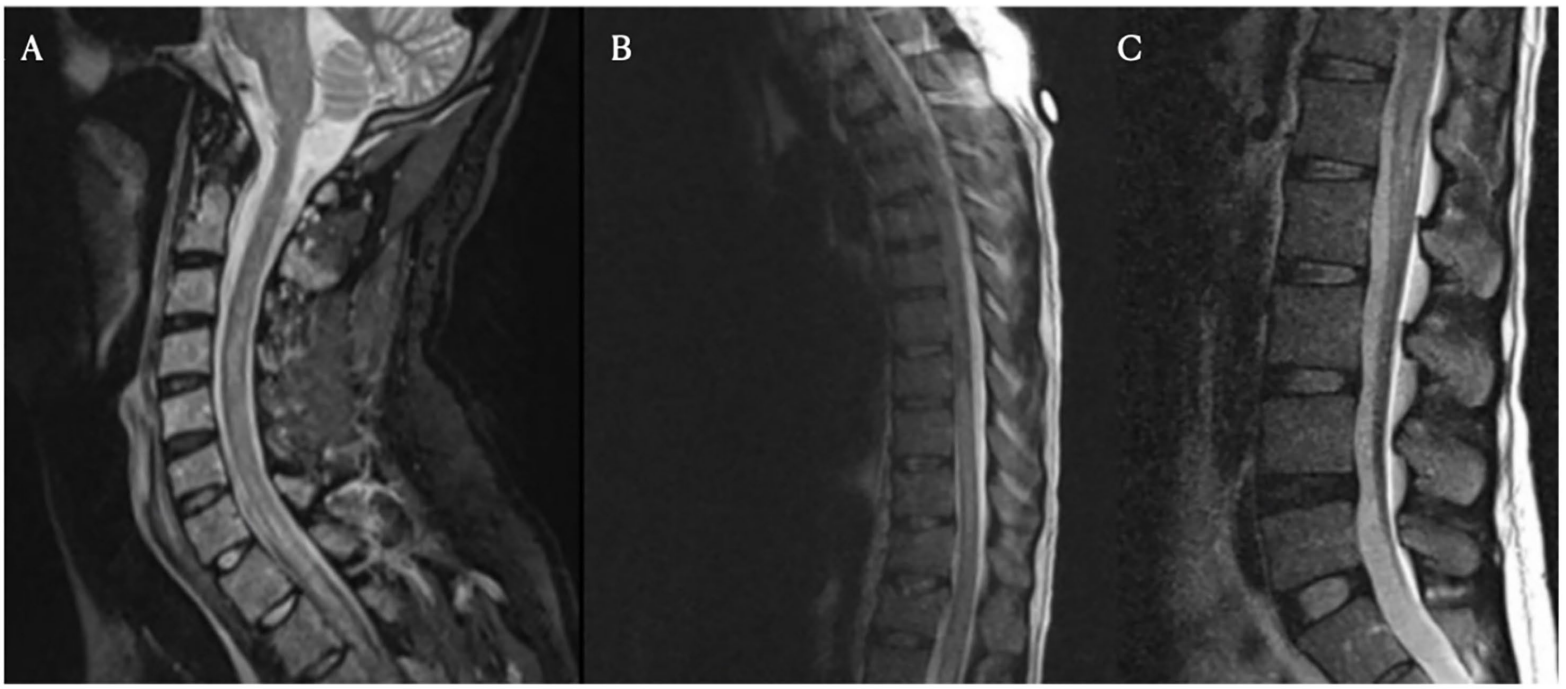
Spinal magnetic resonance imaging on day 10 post-treatment. Cervical (Panel **A**), thoracic (Panel **B**), and lumbar (Panel **C**) spine MRI images obtained on day 10. These images show longitudinally extensive T2 hyperintensity within the spinal cord, most prominent from C7 to T4 and from T8 to the conus medullaris. No significant spinal canal or neural foraminal stenosis is noted at any level.

**Figure 6:**
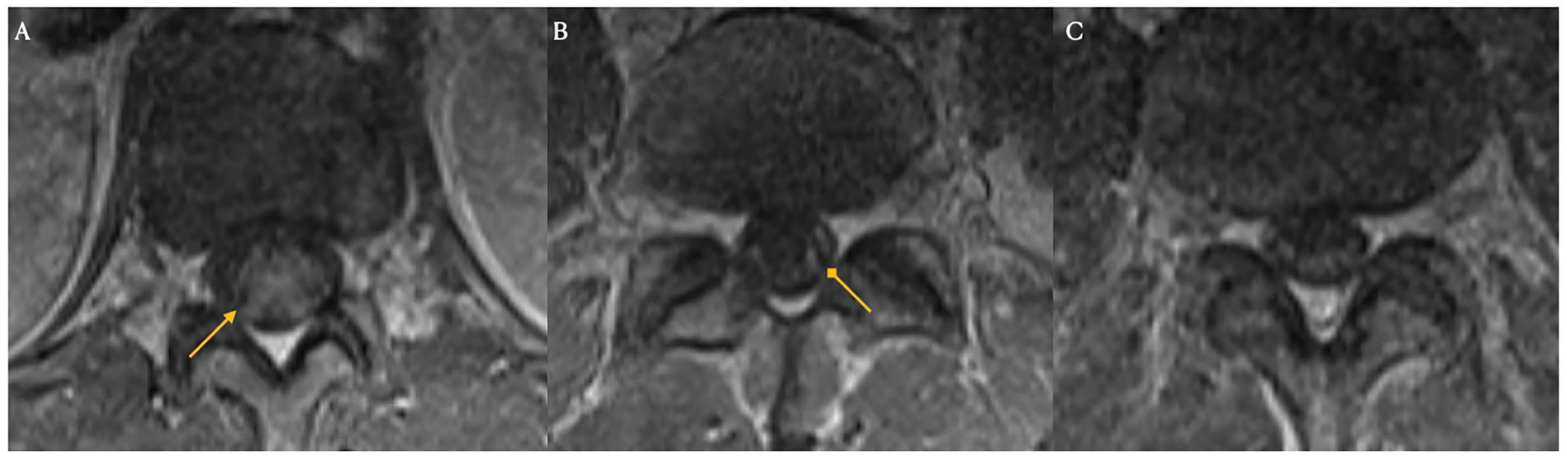
Spinal magnetic resonance imaging on day 21 post-treatment. New clumping and enhancement of the ventral cauda equina nerve roots observed on day 21 after treatment for ICANS and prophylactic IVIG (Panels **A,B,C**).

On day nine following Yescarta infusion, the patient was extubated and noted to have new-onset bilateral lower extremity paresis, areflexia, and sensory loss up to the level of the umbilicus. MRI revealed diffuse T2 hyperintensity throughout the cervical cord, thoracic cord, and conus medullaris (Figure 5). He was empirically initiated on intravenous immunoglobulin (IVIG, 0.4 g/kg for 5 days) while continuing dexamethasone and anakinra therapy. Although neuroimaging showed improvement with this regimen, the patient’s lower extremity weakness persisted without clinical recovery. By day 21, repeat MRI demonstrated complete resolution of the initial brain and spine abnormalities; however, new findings emerged in the conus medullaris, including clumping of the ventral nerve roots (Figure 6). Cerebrospinal fluid (CSF) analysis revealed elevated protein (101 mg/dL), leukocytosis (23 cells/μL), normal glucose (61 mg/dL), and no evidence of malignancy. Infectious cultures, ganglioside antibodies, aquaporin-4, paraneoplastic antibodies, and oligoclonal bands were all negative in the CSF.

Given the persistent neurological deficits and evolving radiographic features, the patient underwent five sessions of plasmapheresis and was additionally treated with eculizumab. Despite comprehensive immunomodulatory therapy, including corticosteroids, anakinra, IVIG, plasma exchange, and eculizumab, there was no significant clinical improvement. The patient was discharged to an acute rehabilitation unit and continues to experience bilateral lower extremity paresis and sensory deficits. He remains under close follow-up with the neurology team.

## Results

Including the present case, a total of 10 cases met the inclusion criteria for evaluation (31–36). Demographic and clinical characteristics, including underlying hematologic malignancy type, CAR T-cell therapy type, and symptom presentation, are summarized in Table 1. The majority of patients (60%) were male, and the underlying malignancies included both multiple myeloma (*n* = 7) and B-cell lymphoma (*n* = 3).

**Table 1:** Summary of demographic and clinical features in reported cases.

Author	Age/Sex	Malignancy	CAR T-cell therapy type	Time to onset (days)	Peripheral nerve neuropathy	Cranial nerve neuropathy	Radiographic findings (MRI brain, spine)	CSF Findings	CRS	ICANS	Intubation	IVIG
Acharya	30/M	B-cell lymphoma (Refractory)	Axicabtagene ciloleucel	+9	Present	Absent	*ICANS*: Leptomeningeal enhancement, diffuse T2/Flair hyperintensities in the pons and cortex *GBS*: conus medullaris with ventral root clumping	Abnormal	Grade1 (+1)	Grade 1 (+3) Grade 3,4 (+4)	Yes (+4)	Yes
Rutenburg (28)	74/M	Multiple myeloma (Refractory)	anti-BCMA CAR-T with ciltacabtagene autoleucel	+13	Present	Present* (LVII)	Abnormal enhancement of the canalicular left facial nerve	Abnormal	Grade 1 (+6)	None	No	Yes
Koch (29)	47/M	B-cell lymphoma (Relapsed)	tisagenlecleucel	+5	Present	Present (RVII)	Abnormal enhancement of the right facial nerve and possibly cauda equina	Abnormal	Grade 1 (+1)	Grade 2 (+5)	Yes (+11)	Yes
Raju (30)	74/M	Multiple myeloma (Refractory)	N/A	+30	Present	Present* (BVII, LVI, RX)	Abnormal enhancement of the canalicular left facial nerve	Abnormal	N/A	N/A	No	Yes
Kuboki (31)	42/F	B-cell lymphoma (Refractory)	tisagenlecleucel	+7	Present	Absent	MRI brain and spine normal	Normal	Grade 1 (+3)	None	No	No
Felipe (32)	56/F	Multiple Myeloma (Consolidation therapy)	anti-BCMA CAR-T	+21	Present	Present (LVII)	Abnormal enhancement of the right facial nerve.	Abnormal	Grade 1 (+2)	None	No	Yes
Miller Patient 1 (33)	66/M	Multiple Myeloma (Refractory)	Idecabtagene vicleucel	+78	Present	Absent	NA.	Abnormal	N/A	N/A	Yes	Yes
Miller Patient 2 (33)	62/M	Multiple Myeloma (Refractory)	Ciltacabtagene autoleucel	+69	Present	Absent	Diffuse spinal nerve root enhancement	Abnormal	N/A	N/A	No	Yes
Miller Patient 3 (33)	66/F	Multiple Myeloma (Refractory)	Ciltacabtagene autoleucel	+14	Present	Present* (BVII)	Abnormal enhancement (L > R) of the facial nerve	Abnormal	N/A	N/A	No	Yes
Miller Patient 4 (33)	81/F	Multiple Myeloma (Refractory)	Ciltacabtagene autoleucel	+25	Present	Present* (BVII)	Abnormal enhancement of the bilateral facial nerves, thickening and clumping of the distal cauda equina roots	Abnormal	N/A	N/A	No	Yes

Demographic and clinical characteristics of case reports, organized by authorship. The indication for therapy is listed in parentheses below the malignancy type. CSF, cerebrospinal fluid analysis; CRS, cytokine release syndrome; ICANS, immune effector cell-associated neurotoxicity syndrome; N/A, not available; NA, not applicable. Symbols: + indicates the number of days post-CAR T-cell therapy when the specified event occurred; * denotes which of two neuropathy types occurred first, if applicable. Nerve abbreviations: LVII, left facial nerve; RVII, right facial nerve; BVII, bilateral facial nerves; LVI, left abducens nerve; RX, right vagus nerve.

Ages across the 10 reported cases ranged from 30 to 81 years, with a mean age of 59.8 years at presentation. Detailed cytokine release syndrome data were available for five cases, all of which were initially diagnosed with grade 1 CRS prior to the onset of neurologic symptoms (31, 32, 34, 36). Among the cases with available timeline data, CRS developed rapidly following CAR T-cell infusion, with onset occurring between 1 and 6 days post-infusion (mean: 2.6 days). Notably, Raju et al. did not report CRS data, and in the series by Miller et al., five of six patients also developed CRS, though specific case-level attribution was not provided (33, 36). Importantly, in all patients described by Miller et al., CRS resolved completely prior to discharge following CAR T-cell therapy (36, 37).

Among the remaining cases with reported CRS, three experienced resolution of symptoms with supportive treatment, including tocilizumab and methylprednisolone, without recurrence or progression at the time neurologic complications developed (31, 34, 35). In contrast, the current case demonstrated a recurrence of grade 1 CRS on day four, despite appropriate initial therapy. A similar clinical course with progression to grade 2 CRS despite treatment was also previously observed by Koch et al. (32).

Both the present case and the case described by Koch et al. developed immune effector cell associated neurotoxicity syndrome. In the Koch et al. case, the patient exhibited simultaneous onset of grade 2 ICANS (disorientation) and GBS-like symptoms on day five, with no ICANS related findings on brain MRI. In contrast, our patient developed grade 1 ICANS on day three, which progressed to grade 3 (aphasia) and grade 4 (seizures) by day four. Brain MRI in our presented case revealed imaging features typical of ICANS, including leptomeningeal enhancement and diffuse T2/FLAIR hyperintensities in the cortex and pons. These findings resolved with treatment using dexamethasone and anakinra. However, following extubation on day nine, the patient developed new-onset neurological deficits and spinal MRI findings consistent with Guillain-Barré syndrome following apparent resolution of ICANS.

In all 10 cases, the onset of GBS occurred with a delay following CAR T-cell therapy, ranging from 5 to 78 days (mean: 27.1 days). When measured from the onset of cytokine release syndrome, the average time to GBS symptom onset was 8.4 days (range: 4–19 days) for cases with available data. All cases involved peripheral neuropathies, characterized primarily by lower extremity weakness and areflexia. Additionally, six patients (60%) presented with cranial nerve involvement, all affecting the facial nerve (31–33, 35, 36). Three of these cases exhibited bilateral facial nerve palsy, one of which had additional involvement of cranial nerves VI and X (33).

Among the six cases with both peripheral and cranial neuropathies, four (reported by Rutenburg et al., Raju et al., and Miller et al.) showed initial cranial nerve deficits followed by delayed onset of peripheral neuropathies, with delays ranging from 2 weeks to 2 months in cases with available data (31, 34, 36, 37). In four of these six cases, MRI findings were limited to abnormal gadolinium enhancement of the facial nerve, despite concurrent peripheral involvement (31–33, 36) In the remaining two cases, Koch et al. and Miller et al. (Patient 4) described similar facial nerve enhancement, with additional clumping and thickening of the cauda equina roots. In contrast, our patient, who presented with only peripheral symptoms, demonstrated distinct conus medullaris abnormalities with ventral nerve root clumping on spine MRI (Table 1).

Nine of the 10 cases (90%) also showed abnormal cerebrospinal fluid profiles, including elevated protein with mild lymphocytosis as well as albuminocytologic dissociation. Notably, Koch et al. identified CAR T-cell expansion within the CSF, with concentrations approximately twice those found in peripheral blood (32). In contrast, Felipe et al. reported no detectable CAR T-cells in the CSF (Table 2) (33). Similarly, nine of the 10 patients received intravenous immunoglobulin (IVIG) therapy. One exception, reported by Kuboki et al., involved a patient with no abnormal findings on MRI or CSF analysis. This individual did not receive IVIG and instead underwent autologous peripheral blood stem cell transplantation due to lymphoma progression and severe cytopenia (35).

**Table 2:** Diagnostic work-up details among GBS cases following CAR T-cell therapy.

Author	Neurologic findings	Lumbar puncture CSF findings	Motor nerve conduction study	Additional workup
Acharya	Bilateral lower extremity paresis, areflexia, sensory loss up to the level of the umbilicus (T10).	Cells: 23, 77% lymphocytes Protein: 1.01 g/L Glucose: 61	N/A	Anti-ganglioside, anti-aquaporin-4 antibodies, paraneoplastic antibodies, oligoclonal bands were negative.
Rutenburg (28)	*Initial*: Left facial palsy with paresthesia, dysphagia, diplopia, and left eye ptosis, right facial palsy *Delayed*: bilateral lower extremity and arm weakness.	Cells: 9, 93% lymphocytes Protein: 0.51 g/L Glucose: 91 mg/dL	N/A	Negative meningo-encephalitis panel and culture
Koch (29)	Facial palsy, incomplete quadriparesis, respiratory muscle weakness.	Cells: 6, 37% CAR-T cells Protein: 0.59 g/L CSF CAR-T 2x peripheral blood IL-6: 6.8 pg./mL	Axonal damage pattern with reduction in compound muscle action potentials, modest reduction in conduction velocity.	Anti-ganglioside and intrathecal antibody screening negative.
Felipe (30)	Proximal paraparesis of the lower limbs with areflexia and left peripheral facial paralysis.	Cells: 11, 75% T lymphocytes Protein: 0.58 g/L Glucose: Normal No CAR-T cells or myeloma cells	N/A	All cultures and viral PCRs were negative.
Raju (31)	*Initial*: Left facial palsy, bilateral lower facial nerve palsy, binocular horizontal diplopia, left CN VI palsy, right CN X palsy *Delayed*: Lower extremity weakness, proximal upper extremity weakness, right wrist drop.	Lymphocytic pleocytosis Protein: 0.51 g/L No IgG synthesis.	Sensorimotor length-dependent axonal neuropathy.	Anti-ganglioside, anti-MAG, anti-aquaporin-4 antibodies were negative.
Kuboki (32)	Motor weakness in bilateral lower extremities.	No abnormalities in total protein, cell count, cytology.	Decline in compound muscle action potential amplitude	N/A
Miller (33) Pt. 1	Areflexia, ascending sensorimotor loss of the bilateral lower extremities followed by autonomic dysfunction, and bulbar/respiratory weakness.	CSF albuminocytologic dissociation	N/A	N/A
Miller (33) Pt. 2	Areflexia, ascending sensorimotor loss of the bilateral lower extremities	CSF albuminocytologic dissociation	Acute axonal sensorimotor polyradiculoneuropathy	N/A
Miller (33) Pt. 3	*Initial*: Bilateral (left > right) facial nerve palsy and dysarthria *Delayed*: Areflexia, ascending sensorimotor loss of the bilateral lower extremities, autonomic dysfunction	CSF albuminocytologic dissociation	Acute axonal sensorimotor polyradiculoneuropathy, low amplitude facial motor responses	N/A
Miller (33) Pt. 4	*Initial*: Bilateral (left > right) facial nerve palsy and dysarthria *Delayed*: Areflexia, ascending sensorimotor loss of the bilateral lower extremities, autonomic dysfunction	CSF albuminocytologic dissociation	N/A	N/A

Summary of findings from included case reports, detailing specific neurological symptoms, cerebrospinal fluid (CSF) analysis results, nerve conduction study findings, and other relevant diagnostic workup, organized by author. N/A, not available.

## Discussion

Guillain-Barré syndrome is a rare but clinically significant complication that should be considered in patients presenting with acute-onset, diffuse weakness following CAR T-cell therapy. Given the relative novelty of CAR T-cell therapy as a cancer treatment and its established potential for neurotoxicity, a comprehensive understanding of its adverse neurological effects is essential. To our knowledge, this is the first review to systematically summarize the clinical features and diagnostic findings of GBS occurring secondary to CAR T-cell therapy.

In the present case, the patient developed multiple neurotoxic complications following Yescarta (axicabtagene ciloleucel) CAR T-cell infusion, including an acute-onset peripheral polyneuropathy consistent with Guillain-Barré syndrome. Notably, he is the youngest reported individual to develop GBS following CAR T-cell therapy and presented with a severe phenotype characterized by rapid onset of CRS and ICANS, which necessitated intubation for airway protection. A comprehensive diagnostic workup was conducted, evaluating several differential diagnoses. While the patient had previously experienced mild neuropathy during treatment with the DA-EPOCH-R regimen, these symptoms had fully resolved prior to CAR T-cell infusion, and there were no clinical signs of the progressive neuropathy typically associated with vincristine exposure. Additionally, no other neurotoxic agents were administered that could account for the abrupt onset of peripheral symptoms.

The patient’s clinical course was also inconsistent with chronic inflammatory demyelinating polyneuropathy (CIDP), given the sudden onset of symptoms, and there was no evidence of preceding multi-organ failure or sepsis to suggest critical illness myopathy (CIM). In the absence of a clear infectious or pharmacologic etiology, the patient’s presentation is most likely attributable to CAR T-cell therapy acting as the inflammatory trigger for the development of GBS. Although detailed case reports of Guillain-Barré syndrome following CAR T-cell therapy are rare, the condition is frequently listed as an adverse event in drug inserts, review articles, and the FDA Adverse Event Reporting System (FAERS) (38–41).

Moreover, GBS has also been documented as a neurologic adverse event associated with other immunotherapies, including adoptive cell therapy (ACT) using tumor-infiltrating lymphocytes, antibody-drug conjugates, and immune checkpoint inhibitors (ICIs) (42–46). Like CAR T-cell therapy, ICIs are linked to a broad spectrum of immune-related adverse events (irAEs), which typically arise within weeks to months of treatment initiation and can affect multiple organ systems (47). Neurologic irAEs include conditions such as aseptic meningitis, encephalitis, myasthenia gravis, and Guillain-Barré-like syndromes (48). While most acute irAEs respond well to corticosteroids, some cases are steroid-resistant, contributing to significant morbidity and mortality (49). Steroid-resistant irAEs, defined as those not improving after high-dose corticosteroids, occur in approximately 10–16% of ICI-treated patients. Management of steroid resistant irAEs is tailored to the specific clinical presentation, with options like IVIG, tocilizumab, or plasmapheresis often used for neurologic complications (50–52). Similarly, chronic irAEs may persist or recur despite immunosuppression, necessitating prolonged therapy and escalation to additional immunomodulatory agents such as intravenous immunoglobulin, plasmapheresis, or biologics. Chronic neurologic irAEs often manifest as myopathies, persistent pain, peripheral neuropathies, or central nervous system pathologies like cognitive impairment (53, 54).

These findings suggest a broader association between immunogenic therapies and aberrant immune activation, leading to peripheral nerve injury. Despite this observed relationship, the underlying neuroimmunologic mechanisms remain poorly understood. Unlike classic GBS, which is driven by molecular mimicry resulting in direct demyelination of peripheral nerves, current evidence does not support a similar mechanism in CAR T-cell–associated GBS. Specifically, there is no indication of peripheral nerve antigen expression or molecular cross reactivity involving CD19 or BCMA targets (55). In addition, anti-ganglioside antibodies, commonly associated with traditional GBS subtypes, were negative in the cases reviewed (Table 2). Therefore, it is unlikely that this pathophysiology is driven by direct, on-target, off-tumor effects involving CD19 or BCMA expression in peripheral nerves. This differs from neurotoxicity, including ICANS, in which there is direct expression of CD19 on pericytes at the blood–brain barrier, leading to loss of membrane integrity (56, 57).

Rather, peripheral neuropathy may be secondary to immune-reactive phenomena following CAR T-cell therapy. Through this mechanism, CRS provokes excessive systemic inflammation, causing indirect peripheral nerve injury by collateral damage. This may explain why GBS has been observed following other immunostimulatory therapies capable of inducing a cytokine storm and is not exclusive to CAR T-cell treatment. This theory is supported by the findings of Koch et al., in which there was marked elevation in inflammatory markers, including C-reactive protein (CRP) and interleukin 6 (IL-6), which peaked on day four, just prior to the onset of neurologic symptoms and ICANS on day five. Similarly, muscle biopsy of this patient revealed perivascular, T-cell dominant lymphocytic infiltrates, neurogenic damage to the muscle, and the presence of CAR T cells in the biopsy, all of which suggest toxicity to vulnerable bystander tissues. In the CARTITUDE-4 trial, patients who developed cranial nerve palsies were likewise found to have elevated levels of interleukins, specifically IL-10 and IL-2Ra, as well as greater CAR T-cell expansion compared to patients without neurologic involvement (58).

The findings within our dataset similarly support this concept, as the majority of the patients who developed GBS had previously presented with CRS; however, only two patients also exhibited ICANS. In ICANS, there is an established relationship between earlier onset and increased severity of CRS and the likelihood of developing ICANS in patients receiving CAR T-cell therapy (32, 59). However, in this cohort, patients with even low-grade CRS went on to develop GBS, and there was no clear temporal relationship between CRS onset and GBS occurrence. Thus, it appears that CRS, regardless of severity, may act as a predisposing factor for non-specific immune dysregulation, as seen in other triggers of GBS like immunizations (60). This may be of particular importance in patients who are genetically susceptible to GBS or immune dysregulation, in whom CRS acts as a second hit (19, 61).

It is equally important to recognize the bidirectional relationship between mental state and neurologic outcomes. In GBS, psychological distress, such as anxiety or depression, is associated with poorer functional recovery and increased disability (62, 63). Given the complex neuroimmune crosstalk, it is worth considering whether emotional distress, particularly in the context of treatment-resistant malignancy, may predispose individuals to the development or worsening of peripheral neuropathies. Psychiatric factors, including chronic stress, depression, and anxiety, are known to modulate immune function and may contribute to the risk of neuroimmunological adverse events, including those induced by ICIs or monoclonal antibody therapies (64–66).

Furthermore, it has been proposed in the literature that patients with certain personality traits or underlying autoimmune diseases who encounter upregulating immunologic triggers—such as CAR-T therapy, infections, etc.—are susceptible to developing psychiatric manifestations of their dysregulated immune response in addition to more physical manifestations like GBS (67, 68). While this phenomenon may occur in patients of any age, it has been best described in the pediatric population, where it is termed PANS (pediatric acute-onset neuropsychiatric syndrome) and PANDAS (pediatric autoimmune neuropsychiatric disorders associated with streptococcal infection) (68). In instances of immunologic hyperactivity, susceptible patients who experience heightened neuropsychiatric symptoms often develop obsessive-compulsive tendencies, hypervigilance, increased neuroticism, and, in some cases, paranoia and/or severe depression with or without suicidality (67–70). Therefore, just as it was important in our case to acknowledge the recent history of CAR-T therapy and its potential for increasing susceptibility to GBS in our patient, it is important to contextualize psychiatric symptoms and determine whether an immune dysregulating process may have triggered them whenever possible, particularly when those immune-dysregulating triggers may be modifiable or treatable so the patient can make an effective recovery from their presenting symptoms.

It is worth noting that in both our case and the case reported by Koch et al., recurrence of CRS and progression to higher-grade CRS, despite timely, appropriate treatment, may indicate a more aggressive or dysregulated immune response, which could increase the risk for severe GBS phenotypes. However, it is important to recognize that, given the high incidence of CRS following CAR T-cell therapy, further investigation is needed to determine whether there is a true mechanistic link or simply a reflection of its prevalence.

As GBS is primarily a clinical diagnosis, recognizing this syndrome in atypical situations such as following CAR T-cell therapy poses a challenge. In our review, a notable pattern emerged in cases involving both peripheral and cranial nerve dysfunction. In the presented cases, not only was concurrent cranial nerve involvement common (60%), but all cases affected the facial nerve. In cases with dual nerve compromise, four of the six cases first presented with cranial nerve symptoms followed by peripheral nerve manifestations in a delayed phase. This differs from the clinical presentation of typical GBS in which peripheral nerve involvement precedes cranial nerve findings, if any are present (28). Similarly, radiographic abnormalities were more frequently identified in cranial nerves, particularly the facial nerve. Commonly, abnormal gadolinium enhancement of the facial nerve was the only imaging finding, even in patients with concurrent diffuse peripheral weakness on examination (66%). While the facial nerve is the most common cranial nerve impacted in standard GBS, this radiographic finding is relatively uncommon and encountered considerably less than peripheral nerve enhancement (71). Miller Fisher syndrome (MFS)—a known GBS variant—also involves cranial nerves such as the facial nerve, but it typically presents with ataxia rather than motor weakness (71). Therefore, GBS following CAR T-cell therapy may represent a distinct clinical and radiologic entity, characterized by early facial nerve palsy followed by peripheral motor deficits, and predominant facial nerve enhancement on imaging. These observations underscore that GBS should not be excluded solely based on the absence of nerve root findings on imaging. Likewise, the onset of cranial nerve palsy in response CAR T-cell therapy may signal impending peripheral neuropathy. This emerging pattern may aid radiologists and clinicians in earlier recognition and diagnosis of this rare but serious complication.

Four of the described patients also presented with peripheral nerve findings on spinal MRI. Similar to typical GBS, these findings included abnormal nerve root enhancement and nerve root thickening. Two cases involved the cauda equina, one affected the conus medullaris, and the last showed diffuse enhancement throughout the cervical, thoracic, and lumbar spine. While GBS usually affects the lumbosacral region, enhancement can extend throughout the cauda equina and conus medullaris (71, 72).

Both our presented case and the case reported by Koch et al. involved patients who developed ICANS and demonstrated radiographic spinal findings consistent with GBS. However, there is currently no evidence supporting a direct link between ICANS and the development of GBS. The co-occurrence of GBS and ICANS appears to be unrelated and most likely reflects individual consequences of excessive cytokine activation. In our case, the patient’s diffuse peripheral weakness persisted despite active treatment and complete radiographic resolution of ICANS associated brain MRI findings. Notably, new MRI spine abnormalities emerged only after ICANS had resolved, suggesting two independent processes. In the case reported by Koch et al., the patient exhibited no ICANS-specific imaging findings and GBS symptoms did not improve with standard ICANS-directed therapy. Additionally, none of the other cases, including the two with peripheral nerve radiologic findings, had preceding central neurotoxicity (36, 37). Thus, the development of GBS appears to be mechanistically distinct from ICANS. Again, further investigation is necessitated to determine whether overlapping immune pathways are involved in these processes.

Albuminocytologic dissociation, defined as an increased protein level (>0.45 g/L) in the absence of elevated white cell count (<50 cells/μL) was seen in 90% of cases (Table 2). While most reports of typical GBS have cell counts <5, many cases here also had a mild lymphocytic pleocytosis, reflecting an inflammatory response within the central nervous system (73, 74). Intravenous immunoglobulin is currently the standard of care for GBS to enhance recovery and reduce disability by modulating the causative autoimmune pathophysiology. IVIG acts to neutralize harmful autoantibodies including anti-ganglioside antibodies, block Fc receptors, inhibit complement activation, and downregulate inflammatory cytokines (75–77). Finally, while IVIG was administered in nearly all reported cases, many patients—including our own—did not demonstrate clear or immediate clinical improvement. In fact, our described patient went on to develop progressive imaging anomalies even after prophylactic IVIG was given (Figure 6), highlighting the uncertainty of IVIG efficacy in this context. If the pathogenesis is not primarily antibody-mediated, the standard therapeutic effect of IVIG may be reduced in this setting, leading to incomplete neurological recovery. These findings underscore the need for further research into the mechanisms underlying CAR-T–associated GBS and the development of targeted treatment strategies beyond conventional GBS management to prevent.

There are multiple limitations to this systemic review. First, the sample size of our study was small given the rarity of GBS following CAR T-cell therapy and the limited case reports available on this topic. Second, our evaluation is based on case reports where there may have been limitations in reporting and oncologic data, and variations in diagnostic testing among cases. Therefore, we cannot conclude any meaningful demographic correlates given the small cohort. These findings may limit the generalizability of our observations. The absence of systematic data collection limits the ability to estimate incidence and prevalence of this specific finding as well as information on long-term prognosis. Future research including multicenter neurotoxicity registries, and prospective studies focusing on serial cytokine measurements are necessary to identify accurate incidence, predisposing biomarker risk factors, and underlying pathogenesis of GBS secondary to CAR T-cell infusion.

## Conclusion

Recognizing and understanding Guillain-Barré syndrome following CAR T-cell therapy is critical given its recent increase in use for refractory malignancies and potentially life threatening consequences. Despite the rarity of reported cases, our review suggests that GBS in this context may present with a distinct clinical and radiographic profile—marked by features consistent with a non-specific inflammatory response rather than classic antibody-mediated pathology. It is essential to identify the critical risk factors for developing Guillain-Barré syndrome after CAR T-cell therapy including individual susceptibility, tumor burden, CAR T-cell therapy type and CAR T-cell therapy amount. While current evidence does not support antigen cross-reactivity between CAR T-cell therapy targets and peripheral nerve components, further investigation into the distinct immunopathology and its mechanistic connection to CRS is necessary. Advancing our understanding of the underlying pathophysiology will be critical for developing targeted approaches to prevent, recognize, and manage this serious complication, as well as for establishing clinical monitoring protocols that enhance patient safety.

## Data Availability

The original contributions presented in the study are included in the article/supplementary material further inquiries can be directed to the corresponding author.

## References

[1] Brudno JN Maus MV Hinrichs CS. CAR T cells and T-cell therapies for cancer: a translational science review. JAMA. (2024) 332:1924–35. doi: 10.1001/jama.2024.19462, PMID: 39495525 PMC11808657

[2] Cao HH Wang LL Geng CK Mao WW Yang LL Ma Y . Therapeutic effects of chimeric antigen receptor T cells (CAR-T) on relapse/refractory diffuse large B-cell lymphoma (R/R DLBCL): a meta-analysis. Eur Rev Med Pharmacol Sci. (2020) 24:4921–30. doi: 10.26355/eurrev_202005_21181, PMID: 32432755

[3] Kim J Cho J Lee MH Yoon SE Kim WS Kim SJ. CAR T cells vs bispecific antibody as third- or later-line large Bcell lymphoma therapy: a meta-analysis. Blood. (2024) 144:629–38. doi: 10.1182/blood.2023023419, PMID: 38696731

[4] Gisselbrecht C Van Den Neste E. How i manage patients with relapsed/refractory diffuse large B cell lymphoma. Br J Haematol. (2018) 182:633–43. doi: 10.1111/bjh.15412, PMID: 29808921 PMC6175435

[5] Tatake IJ Arnason JE. CARs for lymphoma. Best Pract Res Clin Haematol. (2024) 37:101601. doi: 10.1016/j.beha.2025.101601, PMID: 40074511

[6] Schuster SJ Bishop MR Tam CS Waller EK Borchmann P McGuirk JP . Tisagenlecleucel in adult relapsed or refractory diffuse large B-cell lymphoma. N Engl J Med. (2019) 380:45–56. doi: 10.1056/NEJMoa1804980, PMID: 30501490

[7] Neelapu SS Locke FL Bartlett NL Lekakis LJ Miklos DB Jacobson CA . Axicabtagene ciloleucel CAR T-cell therapy in refractory large B-cell lymphoma. N Engl J Med. (2017) 377:2531–44. doi: 10.1056/NEJMoa1707447, PMID: 29226797 PMC5882485

[8] Abramson JS Palomba ML Gordon LI Lunning MA Wang M Arnason J . Lisocabtagene maraleucel for patients with relapsed or refractory large B-cell lymphomas (TRANSCEND NHL 001): a multicentre seamless design study. Lancet. (2020) 396:839–52. doi: 10.1016/S0140-6736(20)31366-0, PMID: 32888407

[9] Berdeja JG Madduri D Usmani SZ Jakubowiak A Agha M Cohen AD . Ciltacabtagene autoleucel, a B-cell maturation antigen-directed chimeric antigen receptor T-cell therapy in patients with relapsed or refractory multiple myeloma (CARTITUDE-1): a phase 1b/2 open-label study. Lancet. (2021) 398:314–24. doi: 10.1016/S0140-6736(21)00933-8, PMID: 34175021

[10] Munshi NC Anderson LD Jr Shah N Madduri D Berdeja J Lonial S . Idecabtagene vicleucel in relapsed and refractory multiple myeloma. N Engl J Med. (2021) 384:705–16. doi: 10.1056/NEJMoa2024850, PMID: 33626253

[11] Karschnia P Dietrich J. Neurological complications of CAR T cell therapy for cancers. Nat Rev Neurol. (2025) 21:422–31. doi: 10.1038/s41582-025-01112-8, PMID: 40562951

[12] Rubin DB Danish HH Ali AB Li K LaRose S Monk AD . Neurological toxicities associated with chimeric antigen receptor T-cell therapy. Brain. (2019) 142:1334–48. doi: 10.1093/brain/awz053, PMID: 30891590

[13] Cosenza M Sacchi S Pozzi S. Cytokine release syndrome associated with T-cell-based therapies for hematological malignancies: pathophysiology, clinical presentation, and treatment. Int J Mol Sci. (2021) 22:7652. doi: 10.3390/ijms22147652, PMID: 34299273 PMC8305850

[14] Freyer CW Porter DL. Cytokine release syndrome and neurotoxicity following CAR T-cell therapy for hematologic malignancies. J Allergy Clin Immunol. (2020) 146:940–8. doi: 10.1016/j.jaci.2020.07.025, PMID: 32771558

[15] Lei W Xie M Jiang Q Xu N Li P Liang A . Treatment-related adverse events of chimeric antigen receptor T-cell (CAR T) in clinical trials: a systematic review and meta-analysis. Cancers (Basel). (2021) 13:3912. doi: 10.3390/cancers13153912, PMID: 34359816 PMC8345443

[16] Li Y Ming Y Fu R Li C Wu Y Jiang T . The pathogenesis, diagnosis, prevention, and treatment of CAR-T cell therapy-related adverse reactions. Front Pharmacol. (2022) 13:950923. doi: 10.3389/fphar.2022.950923, PMID: 36313336 PMC9616161

[17] Gu T Hu K Si X Hu Y Huang H. Mechanisms of immune effector cell-associated neurotoxicity syndrome after CAR-T treatment. WIREs Mech Dis. (2022) 14:e1576. doi: 10.1002/wsbm.1576, PMID: 35871757 PMC9787013

[18] Sterner RC Sterner RM. Immune effector cell associated neurotoxicity syndrome in chimeric antigen receptor-T cell therapy. Front Immunol. (2022) 13:879608. doi: 10.3389/fimmu.2022.879608, PMID: 36081506 PMC9445841

[19] Gust J Hay KA Hanafi LA Li D Myerson D Gonzalez-Cuyar LF . Endothelial activation and blood–brain barrier disruption in neurotoxicity after adoptive immunotherapy with CD19 CAR-T cells. Cancer Discov. (2017) 7:1404–19. doi: 10.1158/2159-8290.CD-17-0698, PMID: 29025771 PMC5718945

[20] Gust J Finney OC Li D Brakke HM Hicks RM Futrell RB . Glial injury in neurotoxicity after pediatric CD19-directed chimeric antigen receptor T cell therapy. Ann Neurol. (2019) 86:42–54. doi: 10.1002/ana.25502, PMID: 31074527 PMC9375054

[21] Grant SJ Grimshaw AA Silberstein J Murdaugh D Wildes TM Rosko AE . Clinical presentation, risk factors, and outcomes of immune effector cell-associated neurotoxicity syndrome following chimeric antigen receptor T cell therapy: a systematic review. Transplant Cell Ther. (2022) 28:294–302. doi: 10.1016/j.jtct.2022.03.006, PMID: 35288347 PMC9197870

[22] Sales C Anderson MA Kuznetsova V Rosenfeld H Malpas CB Roos I . Patterns of neurotoxicity among patients receiving chimeric antigen receptor T-cell therapy: a single-centre cohort study. Eur J Neurol. (2024) 31:e16174. doi: 10.1111/ene.16174, PMID: 38085272 PMC11235605

[23] Fontanelli L Pizzanelli C Milano C Cassano Cassano R Galimberti S Rossini MI . Pre-existing frontal lobe dysfunction signs as predictors of subsequent neurotoxicity in CAR T cell therapy: insights from a case series. Neurol Sci. (2023) 44:3291–7. doi: 10.1007/s10072-023-06841-6, PMID: 37160803 PMC10170036

[24] Pinto SN Liu CJ Nelson MD Jr Bluml S Livingston D Tamrazi B. Neuroimaging of complications arising after CD19 chimeric antigen receptor T-cell therapy: a review. J Neuroimaging. (2023) 33:703–15. doi: 10.1111/jon.13138, PMID: 37327044

[25] Lapidus AH Anderson MA Harrison SJ Dickinson M Kalincik T Lasocki A. Neuroimaging findings in immune effector cell associated neurotoxicity syndrome after chimeric antigen receptor T-cell therapy. Leuk Lymphoma. (2022) 63:2364–74. doi: 10.1080/10428194.2022.2074990, PMID: 35570737

[26] Han MW Jeong SY Suh CH Park H Guenette JP Huang RY . Incidence of immune effector cell-associated neurotoxicity among patients treated with CAR T-cell therapy for hematologic malignancies: systematic review and meta-analysis. Front Neurol. (2024) 15:1392831. doi: 10.3389/fneur.2024.1392831, PMID: 39474369 PMC11518750

[27] Súkeníková L Mallone A Schreiner B Ripellino P Nilsson J Stoffel M . Autoreactive T cells target peripheral nerves in Guillain-Barré syndrome. Nature. (2024) 626:160–8. doi: 10.1038/s41586-023-06916-6, PMID: 38233524 PMC10830418

[28] Shahrizaila N Lehmann HC Kuwabara S. Guillain-Barré syndrome. Lancet. (2021) 397:1214–28. doi: 10.1016/S0140-6736(21)00517-1, PMID: 33647239

[29] Gorson KC. Evolving understanding of Guillain-Barré syndrome pathophysiology and the central role of the classical complement pathway in axonal injury. Front Neurol. (2025) 16:1572949. doi: 10.3389/fneur.2025.1572949, PMID: 40438570 PMC12117664

[30] Wakerley BR Uncini A Yuki N GBS Classification Group. Guillain-Barré and Miller fisher syndromes—new diagnostic classification. Nat Rev Neurol. (2014) 10:537–44. doi: 10.1038/nrneurol.2014.138, PMID: 25072194

[31] Rutenberg D. Guillain-Barré syndrome following anti-BCMA CAR-T cell therapy. Chest. (2023) 164:A6183–4. doi: 10.1016/j.chest.2023.07.3978

[32] Koch C Fleischer J Popov T Frontzek K Schreiner B Roth P . Diabetes insipidus and Guillain- Barré-like syndrome following CAR- T cell therapy: a case report. J Immunother Cancer. (2023) 11:e006059. doi: 10.1136/jitc-2022-006059, PMID: 36690387 PMC9872508

[33] Peña Muñoz F. Neurological complication, possibly related to anti-BCMA CAR-T cell therapy. Presented by the Clinical Hematology Department, Institute Català d’Oncologia–Hospitalet, Barcelona, Spain. The EBMT. Barcelona, Spain: Neurology Department, Neuro-Oncology Unit, Institute Català d’Oncologia–Hospitalet (2024); Physician expert perspective by Velasco, R.).

[34] Raju S Jaffer M Mokhtari S Peguero E. PBC-like variant of GBS associated with CAR-T therapy (P9-11.008). Neurology. (2024) 102:9-11.008. doi: 10.1212/WNL.000000000020544

[35] Kuboki M Umezawa Y Motomura Y Okada K Nogami A Nagao T . Severe motor weakness due to disturbance in peripheral nerves following tisagenlecleucel treatment. In Vivo. (2021) 35:3407–11. doi: 10.21873/invivo.12640, PMID: 34697176 PMC8627748

[36] Miller L Barrell K. Peripheral and cranial neuropathies following CAR-T cell therapy for multiple myeloma: a case series (S16.006). Neurology. (2025) 104:S16.006. doi: 10.1212/WNL.000000000021076

[37] Miller Leah. Peripheral nervous system toxicity following anti-BCMA CAR-T therapy for multiple myeloma: a case series University of Utah Spencer Fox Eccles School of Medicine (2025). Available online at: https://medicine.utah.edu/neurology/grandrounds/video?video=1_wzeku52l

[38] Ciltacabtagene autoleucel [package insert]. CARVYKTI (2022). Available online at: https://www.janssenlabels.com/package-insert/product-monograph/prescribinginformation/CARVYKTI-pi.pdf (Accessed February 28, 2025).

[39] Liu W Lin S Zhu X Yin L Liu Q Lei S . Safety assessment of anti-B cell maturation antigen chimeric antigen receptor T cell therapy: a real-world study based on the FDA adverse event reporting system database. Front Immunol. (2024) 15:1433075. doi: 10.3389/fimmu.2024.1433075, PMID: 39290710 PMC11405296

[40] Zhai Y Yuan L Fang S Liu S Ye X Shi W . Neurotoxicity associated with chimeric antigen receptor T-cell therapy: a real-world study leveraging the FDA adverse event reporting system. Expert Opin Drug Saf. (2024):1–9. doi: 10.1080/14740338.2024.2416542 39410883

[41] Natrajan K Kaushal M George B Kanapuru B Theoret MR. FDA approval summary: ciltacabtagene autoleucel for relapsed or refractory multiple myeloma. Clin Cancer Res. (2024) 30:2865–71. doi: 10.1158/1078-0432.CCR-24-0378, PMID: 38713595 PMC11249607

[42] Joseph J Nathenson MJ Trinh VA Malik K Nowell E Carter K . Guillain-Barre syndrome observed with adoptive transfer of lymphocytes genetically engineered with an NY-ESO-1 reactive T-cell receptor. J Immunother Cancer. (2019) 7:296. doi: 10.1186/s40425-019-0759-x, PMID: 31703609 PMC6842215

[43] Orcurto A Hottinger A Wolf B Navarro Rodrigo B Ochoa de Olza M Auger A . Guillain-Barré syndrome after adoptive cell therapy with tumor-infiltrating lymphocytes. J Immunother Cancer. (2020) 8:e001155. doi: 10.1136/jitc2020-001155 32847987 PMC7451492

[44] Liao B Shroff S Kamiya-Matsuoka C Tummala S. Atypical neurological complications of ipilimumab therapy in patients with metastatic melanoma. Neuro-Oncology. (2014) 16:589–93. doi: 10.1093/neuonc/nou001, PMID: 24482447 PMC3956363

[45] Dubey D David WS Amato AA Reynolds KL Clement NF Chute DF . Varied phenotypes and management of immune checkpoint inhibitor–associated neuropathies. Neurology. (2019) 93:e1093–103. doi: 10.1212/WNL.0000000000008091, PMID: 31405908

[46] Pradeep R Benekli ZN Nair R Malpica L Lee HJ Maples E . Brentuximab associated Guillain-Barré syndrome (BVGBS): case series and a review of pharmacovigilance FDA adverse reporting system (FAERS). Blood. (2024) 144:7776–6. doi: 10.1182/blood-2024-202119

[47] Postow MA Sidlow R Hellmann MD. Immune-related adverse events associated with immune checkpoint blockade. N Engl J Med. (2018) 378:158–68. doi: 10.1056/NEJMra1703481, PMID: 29320654

[48] Di Giacomo AM Bartalini S D'Alonzo V Cerase A Cutarella S Rossi G . Neurological adverse events of ICI therapy: a ten-year comprehensive management from a multidisciplinary team. Eur J Cancer. (2025) 228:115707. doi: 10.1016/j.ejca.2025.115707, PMID: 40839911

[49] Schneider BJ Naidoo J Santomasso BD Lacchetti C Adkins S Anadkat M . Management of immune-related adverse events in patients treated with immune checkpoint inhibitor therapy: ASCO guideline update. J Clin Oncol. (2021) 39:4073–126. doi: 10.1200/JCO.21.01440, PMID: 34724392

[50] Luo J Beattie JA Fuentes P Rizvi H Egger JV Kern JA . Beyond steroids: immunosuppressants in steroid-refractory or resistant immune-related adverse events. J Thorac Oncol. (2021) 16:1759–64. doi: 10.1016/j.jtho.2021.06.024, PMID: 34265432 PMC8464489

[51] Malvaso A Giglio P Diamanti L Gastaldi M Vegezzi E Pace A . Unravelling the acute, chronic and steroid-refractory management of high-grade neurological immune-related adverse events: a call to action. Brain Sci. (2024) 14:764. doi: 10.3390/brainsci14080764, PMID: 39199458 PMC11352216

[52] Ruf T Kramer R Forschner A Leiter U Meier F Reinhardt L . Second-line therapies for steroid-refractory immunerelated adverse events in patients treated with immune checkpoint inhibitors. Eur J Cancer. (2024) 203:114028. doi: 10.1016/j.ejca.2024.114028, PMID: 38652976

[53] Abraham PE Johnson DB. Long-term toxicities of immune checkpoint inhibitors. Drugs. (2025). doi: 10.1007/s40265-025-02243-4 [Epub ahead of print]. PMC12615533 41028650

[54] Rossi S Farina A Malvaso A Dinoto A Fionda L Cornacchini S . Clinical course of neurologic adverse events associated with immune checkpoint inhibitors: focus on chronic toxicities. Neurol Neuroimmunol Neuroinflamm. (2024) 11:e200314. doi: 10.1212/NXI.0000000000200314, PMID: 39298719 PMC11413993

[55] Thompson JA Schneider BJ Brahmer J Zaid MA Achufusi A Armand P . NCCN guidelines® insights: management of immunotherapy-related toxicities, version 2.2024. J Natl Compr Cancer Netw. (2024) 22:582592. doi: 10.6004/jnccn.2024.0057, PMID: 39536465

[56] Parker KR Migliorini D Perkey E Yost KE Bhaduri A Bagga P . Single-cell analyses identify brain mural cells expressing CD19 as potential off-tumor targets for CAR-T immunotherapies. Cell. (2020) 183:126–142.e17. doi: 10.1016/j.cell.2020.08.022, PMID: 32961131 PMC7640763

[57] Pinto SN Krenciute G. The mechanisms of altered blood-brain barrier permeability in CD19 CAR T-cell recipients. Int J Mol Sci. (2024) 25:644. doi: 10.3390/ijms25010644, PMID: 38203814 PMC10779697

[58] van de Donk NWCJ Sidana S Schecter JM Akram M Gallego Perez-Larraya J Rodriguez-Otero P . Clinical experience with cranial nerve impairment in the CARTITUDE-1, CARTITUDE-2 cohorts A, B and C, and CARTITUDE-4 studies of ciltacabtagene autoleucel (cilta-cel). Transplant Cell Ther. (2024) 30:S380–1. doi: 10.1016/j.jtct.2023.12.532

[59] Rubin DB Al Jarrah A Li K LaRose S Monk AD Ali AB . Clinical predictors of neurotoxicity after chimeric antigen receptor T-cell therapy. JAMA Neurol. (2020) 77:1536–42. doi: 10.1001/jamaneurol.2020.2703, PMID: 32777012 PMC7418044

[60] Israeli E Agmon-Levin N Blank M Chapman J Shoenfeld Y. Guillain-Barré syndrome—a classical autoimmune disease triggered by infection or vaccination. Clin Rev Allergy Immunol. (2012) 42:121–30. doi: 10.1007/s12016-010-8213-3, PMID: 20890797

[61] Zhao Y Zhu R Tian D Liu X. Genetic polymorphisms in Guillain-Barré syndrome: a field synopsis and systematic meta-analysis. Autoimmun Rev. (2020) 19:102665. doi: 10.1016/j.autrev.2020.102665, PMID: 32949724

[62] Li CMF Wong S Fabiano N . Systematic review: mental health outcomes in Guillain-Barré syndrome and chronic inflammatory demyelinating polyneuropathy. Can J Neurol Sci. (2025):1–10. doi: 10.1017/cjn.2025.10107 40485150

[63] Khan F Pallant JF Ng L Bhasker A. Factors associated with long-term functional outcomes and psychological sequelae in Guillain-Barré syndrome. J Neurol. (2010) 257:2024–31. doi: 10.1007/s00415-010-5653-x, PMID: 20625757

[64] Accorroni A Nencha U Bègue I. The interdisciplinary synergy between neurology and psychiatry: advancing brain health. Clin Transl Neurosci. (2025) 9:18. doi: 10.3390/ctn9010018

[65] Santos JC Pyter LM. Neuroimmunology of behavioral comorbidities associated with cancer and cancer treatments. Front Immunol. (2018) 9:1195. doi: 10.3389/fimmu.2018.0119567 29930550 PMC6001368

[66] Gorson KC Ropper AH Muriello MA Blair R. Prospective evaluation of MRI lumbosacral nerve root enhancement in acute Guillain-Barré syndrome. Neurology. (1996) 47:813–7. doi: 10.1212/wnl.47.3.813, PMID: 8797486

[67] Kang W Malvaso A. Associations between personality traits and areas of job satisfaction: pay, work itself, security, and hours worked. Behav Sci (Basel). (2023) 13:445. doi: 10.3390/bs13060445, PMID: 37366697 PMC10295380

[68] Gamucci A Uccella S Sciarretta L D'Apruzzo M Calevo MG Mancardi MM . PANDAS and PANS: clinical, neuropsychological, and biological characterization of a monocentric series of patients and proposal for a diagnostic protocol. J Child Adolesc Psychopharmacol. (2019) 29:305–12. doi: 10.1089/cap.2018.0087, PMID: 30724577

[69] Cocuzza S Maniaci A La Mantia I Nocera F Caruso D Caruso S . Obsessive-compulsive disorder in PANS/PANDAS in children: in search of a qualified treatment—a systematic review and metanalysis. Children. (2022) 9:155. doi: 10.3390/children9020155, PMID: 35204876 PMC8869780

[70] Mazza MG De Lorenzo R Conte C Poletti S Vai B Bollettini I . Anxiety and depression in COVID-19 survivors: role of inflammatory and clinical predictors. Brain Behav Immun. (2020) 89:594–600. doi: 10.1016/j.bbi.2020.07.037, PMID: 32738287 PMC7390748

[71] Noioso CM Bevilacqua L Acerra GM Della Valle P Serio M Vinciguerra C . Miller fisher syndrome: an updated narrative review. Front Neurol. (2023) 14:1250774. doi: 10.3389/fneur.2023.1250774, PMID: 37693761 PMC10484709

[72] Byun WM Park WK Park BH Ahn SH Hwang MS Chang JC. Guillain-Barré syndrome: MR imaging findings of the spine in eight patients. Radiology. (1998) 208:137–41. doi: 10.1148/radiology.208.1.9646804, PMID: 9646804

[73] Al-Hakem H Doets AY Stino AM . CSF findings in relation to clinical characteristics, subtype, and disease course in patients with Guillain-Barré syndrome. Neurology. (2023) 100:e2386–97. doi: 10.1212/WNL.0000000000207282, Erratum in: *Neurology*. 2023;101(13):592. doi:10.1212/WNL.0000000000207874 37076309 PMC10256127

[74] Fokke C van den Berg B Drenthen J Walgaard C van Doorn PA Jacobs BC. Diagnosis of Guillain-Barré syndrome and validation of Brighton criteria. Brain. (2014) 137:33–43. doi: 10.1093/brain/awt285, PMID: 24163275

[75] Sater RA Rostami A. Treatment of Guillain-Barré syndrome with intravenous immunoglobulin. Neurology. (1998) 51:S9–S15. doi: 10.1212/wnl.51.6_suppl_5.s9, PMID: 9851724

[76] Zhang G Massaad CA Gao T Pillai L Bogdanova N Ghauri S . Sialylated intravenous immunoglobulin suppress antiganglioside antibody mediated nerve injury. Exp Neurol. (2016) 282:49–55. doi: 10.1016/j.expneurol.2016.05.020, PMID: 27208700 PMC5351292

[77] Morse BA Motovilov K Brode WM Tee FM Melamed E. A review of intravenous immunoglobulin in the treatment of neuroimmune conditions, acute COVID-19 infection, and post-acute sequelae of COVID-19 syndrome. Brain Behav Immun. (2025) 123:725–38. doi: 10.1016/j.bbi.2024.10.006, PMID: 39389388

